# Field evaluation of a real time loop-mediated isothermal amplification assay (RealAmp) for malaria diagnosis in Cruzeiro do Sul, Acre, Brazil

**DOI:** 10.1371/journal.pone.0200492

**Published:** 2018-07-11

**Authors:** Giselle Maria Rachid Viana, Luciana Silva-Flannery, Danielle Regina Lima Barbosa, Naomi Lucchi, Suiane Costa Negreiros do Valle, Samela Farias, Nayara Barbalho, Paola Marchesini, Juliana Chedid Nogaredi Rossi, Venkatachalam Udhayakumar, Marinete Marins Póvoa, Alexandre Macedo de Oliveira

**Affiliations:** 1 Instituto Evandro Chagas–IEC/SVS/MS, S/N° Bairro: Levilândia, Ananindeua, Pará, Brasil; 2 Malaria Branch, Division of Parasitic Diseases and Malaria, Centers for Global Health, Centers for Disease Control and Prevention, Atlanta, GA, United States of America; 3 Secretaria Estadual de Saude do Acre—Hemonúcleo Cruzeiro do Sul. Manuel Terças, Cruzeiro do Sul–Acre—Brasil; 4 Coordenação Geral do Programa Nacional de Controle da Malária e Doenças Transmitidas pelo *Aedes*- CGPNCM Setor Comercial Sul, Edifício Principal, Brasília/DF, Brasil; Academic Medical Centre, NETHERLANDS

## Abstract

Conventional molecular methods, such as nested polymerase chain reaction (PCR), are very sensitive for detection of malaria parasites, but require advanced laboratory equipment and trained personnel. Real-time loop-mediated isothermal amplification (RealAmp), a loop-mediated isothermal amplification-based molecular tool (LAMP), facilitates rapid target amplification at a single temperature setting, reducing the need for sophisticated equipment. We evaluated the performance of a field-adapted RealAmp assay for malaria diagnosis in Cruzeiro do Sul, Acre State, Brazil, a remote area in Brazil with limited laboratory capabilities. We enrolled 1,000 patients with fever (axillary temperature ≥ 37.5 C) or history of fever in last 24 h presenting for malaria diagnosis from February through June 2015. DNA was extracted from dried blood spots using a boil and spin method (heat treatment) at the sample processing site, and also using commercial kits at a Brazilian national reference laboratory. RealAmp was performed for *Plasmodium* genus, *P*. *falciparum*, and *P*. *vivax* identification. In addition, Giemsa-stained blood smears were prepared and examined by two independent well-trained study microscopists. A combination of Real-time PCR and nested PCR was used as reference test. The sensitivity and specificity of RealAmp in the field site laboratory were 94.1% (95% confidence interval [CI]: 90.1–96.8) and 83.9% (95% CI: 81.1–86.4), respectively. The sensitivity and specificity of local microscopy were 87.7% (95% CI: 82.6–91.7) and 98.9% (95% CI: 97.8–99.4), respectively, while study microscopy showed sensitivity of 96.4% (95% CI: 93.0–98.4) and specificity of 98.2% (95% CI: 97.0–99.0). None of the three tests detected 20 *P*. *falciparum* and *P*. *vivax* mixed infections identified by the reference test. Our findings highlight that it is possible to implement simple molecular tests in facilities with limited resources such as Cruzeiro do Sul in Brazil. RealAmp sensitivity was similar to that of microscopy performed by skilled professionals; both RealAmp and study microscopy performed poorly in detection of mixed infection. Attempts to develop and evaluate simpler molecular tools should continue, especially for the detection of malaria infection in remote areas.

## Introduction

In general, molecular methods are more sensitive than microscopy and rapid diagnostic tests (RDTs) to detect malaria infection, particularly in cases of low-density parasitemia, which are known to be capable of continuing malaria transmission, and mixed infections [1,2]. However, conventional molecular methods, such as polymerase chain reaction (PCR) and real-time PCR, can be challenging to implement. They demand high investments in laboratory equipment and trained personnel, restricting their use to reference laboratories [3–5].

The need for simpler molecular-based methods for use in settings that have limited laboratory infrastrucure and lack highly skilled technicians and sophisticated equipment led to the development of the loop-mediated isothermal amplification (LAMP) method [6]. Different platforms of the LAMP assay for malaria parasite detection have since been developed and evaluated [7–10]. One of these is the RealAmp assay that combines the LAMP technique with real-time detection of fluorescence as a read out [7]. RealAmp allows the detection of the genus *Plasmodium*, and *P*. *vivax* and *P*. *falciparum* species. It presents a great potential to extend the use of molecular methods even to remote health posts with limited laboratory infrastrucure. However, to date, there are few studies evaluating RealAmp under these settings using clinical samples [11]. Here, we evaluated the sensitivity and specificity of RealAmp when performed in a remote malaria-endemic area of the Brazilian Amazon with limited laboratory infrastrucure by personnel without previous training in molecular techniques.

## Material and methods

### Collection site

Sample collection took place at a malaria diagnostic post in Cruzeiro do Sul, Acre state, Brazil. In Brazil, malaria diagnostic posts provide free malaria diagnosis and treatment. We chose the diagnostic post located in the emergency department of the Regional Hospital of Juruá. This diagnostic post, which is open 24 hours a day/7 days a week, serves approximately 62 patients per day and has the highest volume of patients in the city. We included patients ≥7 years old (excluding pregnant women) with documented fever (axillary temperature >37.5^o^ C) or history of fever in the previous 24 hours. Patients under 7 years of age were excluded because they were accompanied by those who were not able to sign the permission from guardians instrument and appeared to have nutritional and growth deficits. After written informed consent was provided for patients 18 years old, or permission from guardians and patient assent from those <18 years old, we collected demographic and clinical information using standard forms and collected blood samples to be tested for malaria.

### Sample size

We had resources to enroll a total of 1,000 patients as part of this evaluation. Considering a historical trend of 6% and 20% of tested patients in Acre state to be positive for *P*. *falciparum* and *P*. *vivax*, respectively, [12] this sample size would be able to detect a change in sensitivities of at least 14% and 10% from baseline sensitivities of 85% and 90%, respectively, for *P*. *falciparum*. Similarly, for the expected 20% incidence of *P*. *vivax*, we would be able to detect a change in sensitivities of at least 8%, 7%, and 4% from baseline sensitivities of 85%, 90%, and 95%, respectively. All these analyses have at least 80% power and an alpha error <0.05. Power analysis calculations for sensitivity were done considering one-sided tests and used NCSS 11 software (NCSS LLC, Kaysville, USA).

### Sample preparation

We collected blood samples at the same time patients were having their blood collected by the diagnostic post staff. Blood was collected by intravenous puncture since it was required for the different purposes of the study. The blood was used to prepare two sets of thick and thin blood smears and two filter paper cards with four blood spots each (Whatman® FTA Elute Cards) in the form of four blood spots. Microscopy was performed at the diagnostic post according to usual procedures (“local microscopy”); these results were recorded by study staff.

### Study microscopy

Slides for a confirmatory Giemsa-stained thick and thin blood smears were prepared in accordance with recommendations from the Brazilian Ministry of Health [13]. The thin smear was used to confirm species identification and the thick smear was used to estimate parasite density. To ensure the quality of staining, all stock solutions and reagents were prepared and distributed by Instituto Evandro Chagas prior to initiating the evaluation. In addition, training on preparation and staining of slides was conducted by Evandro Chagas laboratory staff during evaluation implementation as well as during bimonthly supervision visits.

As per routine procedure, parasite density was estimated by counting the number of asexual parasites per 200 white blood cells (WBCs) in the thick smear and assuming a WBC count of 6,000 WBC/μL. If the parasite count was < 100 parasites at the 200 WBC mark, counting was continued until 500 WBCs had been counted. If no asexual forms had been found when WBC count had reached 500, the count was continued until 1,000 WBCs had been counted to confirm a negative result. Gametocytes were also counted and the number of gametocytes/ μL was estimated in the same manner.

Study blood smears were examined independently and in a blinded fashion by two microscopists in Cruzeiro do Sul. When the two readings differed in species diagnosis or by more than 50% in asexual parasite density, smears were re-examined by a third, independent, microscopist at Instituto Evandro Chagas. Asexual parasite density was calculated by averaging the counts of the two concordant microscopists using geometric mean, while gametocytemia density was calculated by the arithmetic means of the two concordant readings. The use of geometric and arithmetic means was based on the expected distribution of these values in previous evaluations.

### Molecular laboratory conditions

All molecular procedures were carried out in a small laboratory in Cruzeiro do Sul, Acre state, Brazil, approximately 3 miles from the sample collection site. This was an improvised building with limited laboratory infrastructure, herein referred to as the field site. The study staff lacked previous experience in molecular techniques and received training for two weeks on performing the RealAmp assay and on the heat-treatment DNA extraction method before the study started.

### Heat-treatment DNA extraction from FTA-cards

In the field site, one single circle of the filter paper was cut into small pieces and placed into a 1.5mL tube, and 500μL of sterile double-distilled water was added. The mix was vortexed three times for 15 seconds each. The supernatant was aspirated and discarded. Forty μL of double distilled water was added and the contents were heated at 95°C for 30 minutes using a heating block. The tube was then spun at 3,200 rpm for 5 minutes and 2 μL of the supernatant was used for RealAmp method in the field site (Cruzeiro do Sul, Acre) on the same day of the heating procedure.

### Conventional DNA extraction

At Instituto Evandro Chagas in Para State, DNA was extracted from the filter paper using a QIAmp DNA Mini Kit following manufacturer’s instructions (Qiagen, Valencia, CA-Qiagen method). The extracted DNA was aliquoted and stored at -20° C until further use in real time PCR and nested PCR.

### RealAmp method

To simplify the RealAmp procedures, a two-component ready-to-use in-house reaction buffer was used for each primer set. Component A contained all the necessary reaction components (buffer, the fluorescent dye SYTO-9, and the primers) except the Bst polymerase; Component B contained the Bst polymerase. Three different RealAmp assays were performed: *Plasmodium* genus, *P*. *falciparum* and *P*. *vivax* assays. To perform the RealAmp assay, 10 μL of Component A was mixed with 0.5 μL of Component B, this was mixed carefully and 2 μL of DNA sample was added. DNA amplification was carried out using the commercial ESE-Quant Tube Scanner (ESE Gmbh, Stockach, Germany) set to collect fluorescence signals at 1-minute intervals for 90 minutes. Each reaction run included positive controls (for *Plasmodium* genus, *P*. *falciparum*, or *P*. *vivax* assays) and a no-template negative control (distilled water). A RealAmp result was considered positive if an increase above the background noise in fluorescence was detected (amplification plot) and negative if there was no fluorescence (flat line). Twenty percent of the samples were randomly selected and retested at CDC Malaria laboratory to evaluate concordance between the laboratories.

### Real-time PCR method

Real-time PCR was conducted using previously described methodology [4] at Instituto Evandro Chagas. Commercially available TaqMan Universal master mix (Applied Biosystems) using primers Plasmo Forward and Plasmo Reverse, and four TaqMan probes corresponding to *P*. *falciparum*, *P*. *vivax*, *P*. *malariae*, and *P*. *ovale* were added to the Master Mix. Amplification and detection of the amplified product were performed on a StepOne Plus Real Time (Applied Biosystems). The following cycling conditions were used for the PCR: an initial step at 50° C for 2 min, 95° C for 10 min, and 45 cycles of 95° C for 15 s and 60° C for 1 min. Positive (known *P*. *falciparum*, *P*. *vivax*, *P*. *malariae*, and *P*. *ovale* positive samples) and negative (no template/DNA) control samples were included with each run.

Each reaction of the Real-time PCR was performed in duplicate; a cut-off *Ct* value of 40 was used to determine a whether samples were positive (*Ct*<40) or negative (*Ct* >40.5). In case of disagreement between duplicates, a third reaction was carried out which was used as a tie-breaker. Twenty percent of all runs were repeated at CDC to check for concordance between the laboratories.

### Nested PCR

Nested PCR was conducted in cases of disagreement between study microscopy and the real-time PCR results. Nested PCR was performed with primers and cycling conditions as previous described [14]. Reactions were performed in 20 μL total volume containing 1X buffer, 2.5 mM MgCl_2_, 200 mM dNTPs, 200 nM primers, and 1.25 units of Taq Polymerase (New England Biolabs, Ipswich, MA). The PCR amplification product was analyzed using gel electrophoresis (2% gel). Reference test was either real-time PCR or nested PCR, when the latter was performed.

### Data analysis

Sensitivity and specificity, with 95% confidence intervals (95% CI), of RealAmp and study microscopy results were calculated based on comparison to the reference test results. Sensitivity and specificity of the microscopic diagnosis at malaria diagnostic posts (local microscopy) were also calculated.

### Ethics statement

Samples used in this study were obtained as part of an evaluation in Cruzeiro do Sul, Acre, Brazil. This study was approved by both Instituto Evandro Chagas (CEP/IEC/SVS/MS #04/2015) and the CDC Institutional Review Board (protocol #6619). Informed written consent was obtained from each participant ≥18 years old; permission from caregivers was obtained for participants <18 years old.

## Results

Study staff training on the RealAmp assay in the field site was completed within two weeks. Training was focused on how to process the DNA extraction by heat treatment method, to prepare the RealAmp reaction, and finally to use the tube scanner machine. The RealAmp components kits format adopted in this evaluation reduced preparation time for the assay, and was demonstrated to be stable throughout the study period with no drop-off in DNA amplification efficiency.

A total of 1,000 samples were collected from patients in Cruzeiro do Sul, Acre State from January through June 2015.The median age of participants was 29.5 years (range: 7–88). Among participants for whom information on sex was available, the majority was male (551 patients, 55%). Two hundred and fifty-two (25.0%) had fever documented at enrolment. Study microscopy yielded 226 positive results, seven with parasite density <50, 20 between 50 and 200 parasites/uL, 28 between 200 and 500 parasites/uL, and finally 171 with parasite density ≥500 parasites/μL among microscopy positive samples. The geometric mean density asexual parasitemia by study microscopy among the patients included in our study was 1,735 parasites/uL (range: 21–19,857 parasites/uL), while the arithmetic mean gametocytemia was 45.9 parasites/uL (range: 0–1,092 parasites/uL).

As summarized in Table 1, the reference test found 48 samples positive for *P*. *falciparum*, 152 positive for *P*. *vivax*, 20 mixed infection (*P*. *falciparum* and *P*. *vivax*), and 780 negative. RealAmp performed in the field site identified 55 samples as *P*. *falciparum* positive, 181 *P*. *vivax*, and 761 negative. Three samples were found to be genus positive but both *P*. *falciparum* and *P*. *vivax* negative by the RealAmp method.

**Table 1:** Results of tests for malaria among samples collected in Cruzeiro do Sul, Acre state (n = 1,000).

Species identified	Local microscopy (n)	Study microscopy (n)	RealAmp field (n)	Reference test (n)
***P*. *falciparum***	42	56	55	48
***P*. *vivax***	153	169	181	152
**Mixed infection**	0	1	0	20
**Species unidentified**	1	0	3	0
**Negative**	804	774	761	780

Mixed infections were not detected by RealAmp, or by microscopy either locally or by our study team. Of the 20 mixed infections detected by the reference test, RealAmp detected seven as *P*. *vivax*, nine as *P*. *falciparum*, one as negative; three samples were genus positive but with species unidentified. Local microscopy detected nine as *P*. *vivax*, nine as *P*. *falciparum*, one as negative, and one as positive for malaria but with species unidentified. Finally, study microscopy identified nine as *P*. *vivax* and eleven as *P*. *falciparum* (Table 2).

**Table 2:** Results of different tests for malaria in samples confirmed to have mixed infections in Cruzeiro do Sul, Acre state (n = 20).

Method	*P*. *falciparum* (n)	*P*. *vivax* (n)	Negative (n)	Mixed (n)	Species unidentified (n)
**Local microscopy**	9	9	1	-	1
**Study microscopy**	9	10	-	1	-
**RealAmp field**	9	7	1	-	3

Study microscopy showed 96.4% (95% CI: 93.0–98.4) sensitivity and 98.2% (95% CI: 97.0–99.0) specificity in comparison with the reference test. The performance of RealAmp in field showed 94.1% (95% CI: 90.1–96.8) sensitivity and 83.9% (95% CI: 81.1–86.4) specificity when compared to the reference test (Table 3). The sensitivity of RealAmp varied from 94.4 to 94.7% for parasite densities of >50, >200, and >500 parasites/μL. The sensitivity was 89.5% (95% CI: 78.5–96.0) when analysis was restricted to those with parasite density <500 parasites/μL. The sensitivity and specificity of local microscopy were 87.7% (95% CI: 82.6–91.7) and 98.9% (95% CI: 97.8–99.4), respectively.

**Table 3:** Sensitivity and specificity of the laboratory assays among samples collected in Cruzeiro do Sul, Acre state (n = 1,000).

Method	Sensitivity %(95% CI%)	Specificity %(95% CI)
**Local microscopy**	87.7 (82.6–91.7)	98.9 (97.8–99.4)
**Study microscopy**	96.4(93.0–98.4)	98.2 (97.0–99.0)
**RealAmp field**	94.1(90.1–96.8)	83.9 (81.1–86.4)

## Discussion

Simpler molecular methods for use under field conditions began to be developed in the last decade [6,7,10,15–17], awakening to the possibility of using molecular methods even in facilities with limited laboratory infrastructure. Isothermal amplification assays such as LAMP are well suited for this use, as they do not need thermalcyclers to amplify the DNA and simpler DNA extraction methods can be used. Our evaluation showed the feasibility of deploying the RealAmp method in a field setting using heat-treatment DNA extraction and a two-component ready-to-use reaction kit. We observed a sensitivity of 94.1%, and a specificity of 83.9% using the RealAmp compared to 96.4% and 98.2% for the study microscopy. A previous study comparing RealAmp with PCR in field settings in India and Thailand showed similar sensitivities, but had a much smaller sample size [11]. Our study is the first one to use a large sample size (1,000 samples) to evaluate the performance of RealAmp in a malaria endemic region where both *P*. *falciparum* and *P*. *vivax* are endemic.

We also showed that RealAmp can be utilized by non-expert staff. Although training was required before this evaluation could be implemented, the assay can be performed by malaria diagnostic technicians even in remote settings with limited laboratory infrastructure. In addition, the RealAmp components kits format adopted in this evaluation reduced preparation time for the assay, without negative consequences for assay performance, in accordance to what was also observed by other authors [11].

Some limitations of the RealAmp platform used in this study include the fact that only six samples can be evaluated per run, limiting its utility for large scale studies. However, it is possible to design other prototypes of the tube scanner that support testing greater number of samples [11]. In addition, RealAmp does not allow the quantification of parasite density. This is a limitation of the LAMP assays in general, as currently there is no quantitative LAMP assay available. Finally, because our study was conducted only in a unique municipality, Cruzeiro do Sul (Acre), and the collection of the isolates was non-random, this study may not be generalizable.

The identification of mixed infections is critical for case management and in epidemiological studies of malaria. Mixed infections were not detected by either the RealAmp or the local and study microscopy in our study, excepted for a unique case detected by study microscopy. This was unexpected for the RealAmp assay given that it is a molecular assay; the primers utilized in our RealAmp assay may require further improvements to enable the detection of mixed infections when present. Previous results demonstrated that the genus-specific RealAmp primers had lower limits of detection than the species-specific primers [7]. This could explain the three genus positive samples in which we could not determine the species. Efforts should be made to review and improve protocols for the detection of mixed malaria infections. However, identification of mixed infections is a great challenge even for expert microscopists since the differentiation of the young forms of trophozoites among the *Plasmodium* species is difficult [18].

Although the current recommendations are to use microscopy or RDTs for detection of malaria parasites before treatment in clinical management, a molecular assay such as RealAmp can be useful in urban centers to increase the diagnostic capacity, besides the offer of microscopy and RDTs. Beyond the risk of decreased sensitivity using microscopy, which has a limit of 50 parasites/μL, some HRP2-based RDTs are failing due to the deletion of the hrp2 and/or hrp3 genes in some *P*. *falciparum* isolates [19,20]. Molecular methods have the potential to detect low parasite densities and the development of such methods that can be used as part of routine care is much desired. While conventional PCR-based methods appear to be the most sensitive assays currently available, the requirement for DNA extraction, sophisticated equipment for amplification, and result interpretation and analysis limit their use to reference laboratories.

In our study, local microscopy obtained a sensitivity of 87.7% and specificity of 98.9%, while study microscopy obtained a sensitivity value of 96.4% and specificity of 98.2% when compared with the reference test. Possible explanations for these differences are better quality staining materials and protocols and also better compliance by the study microscopists to the malaria microscopy protocols recommended by the Brazilian Ministry of Health [13,21,22]. The sensitivity of thick smears depends on the experience of the microscopist and, even when evaluated at qualified centers or reference laboratory, thick smears cannot always precisely differentiate among *Plasmodium* species with similar morphology, which is the case for *P*. *vivax* and *P*. *ovale* [23]. Moreover, *P*. *knowlesi* infections can be misdiagnosed by microscopy as *P*. *malariae* due to their morphological similarities [24].

Although the specificity of study microscopy was higher than that of RealAmp, our evaluation demonstrates that RealAmp using simple sample preparation, heat-treatment, has sensitivity similar to that of good quality microscopy. Novel formats of the tube scanner that can support testing of a greater number of samples will offer a screening tool for surveillance studies required for malaria control and elimination. However, further improvements will be required to make the RealAmp assay an alternative point-of-care diagnostic and an alternative molecular assay in resource-limited settings. These include the use of lyophilized reagents format that would remove the need for a cold chain and minimize the preparation time for the reaction components, and improving the assays to enable the detection of mixed infections. Indeed, such improvements have been made in the much newer malaria LAMP platforms such as the FIND/Eiken high-throughput LAMP platform [25] or the *Illunigene* Malaria LAMP assay [26].

In parallel to the LAMP platforms, another molecular techniques are being currently developed, as nucleic acid lateral flow immunoassay (NALFIA) and PCR Nucleic Acid Lateral Flow Immunoassay (db-PCR-NALFIA), that has the advantage of avoiding DNA extraction which is of great interest for techniques intended to be used in the field setting [27,28]. Although in the laboratory validation, the sensitivity and specificity of the db-PCR-NALFIA to pan/*P*. *falciparum* assay were 100% for both parameters, and 100% and 97.5% to pan/*P*. *vivax* assay, respectively, this scenario is not the same in clinical samples. Since in the prospective field evaluation of the pan/*P*. *falciparum* assay performed in Kenya, the sensitivity decreased to 84.5% and specificity to 85.4%, respectively, when compared with real-time quantitative PCR as a reference standard [28]. Therefore, while our findings and other investigations highlights to the possibility of implementing simple, sensitive and specific molecular methods to remote areas, improvements are still required to make these tools technically and operationally viable to the point-of-care diagnosis. Thus, studies for the development and evaluation of simple, ultra-sensitive and specific molecular assays for precise malaria detection should continue, especially for use of these tools in remote areas [25–28].

## Conclusions

Our study demonstrates that it is possible to implement simpler molecular tests at point-of-care centers with limited laboratory infrastructure such as Cruzeiro do Sul and, with further improvements to the available malaria LAMP assays and this technology can become an alternative to PCR-based molecular tests where these more sensitive assays are required.

## Product disclaimer

Use of trade names and commercial sources is for identification only and does not imply endorsement by the U.S. Department of Health and Human Services.

## References

[1] Coleman RE, Sattabongkot J, Promstaporm S, Maneechai N, Tippayachai B, Kengluecha A, et al Comparison of PCR and microscopy for detection of asymptomatic malaria in a *Plasmodium falciparum/ vivax* endemic area in Thailand. Malar J. 2006; 5:121 doi: 10.1186/1475-2875-5-121 17169142 10.1186/1475-2875-5-121 PMC1716172

[2] Boonma P, Christensen PR, Suwanarusk R, Price RN, Russel B, Lek-Uthai U. Comparison of three molecular methods for the detection and speciation of *Plasmodium vivax* and *Plasmodium falciparum*. Malar J. 2007; 6:124 doi: 10.1186/1475-2875-6-124 17868467 10.1186/1475-2875-6-124 PMC2020467

[3] Kimura M, Kaneko O, Liu Q, Zhou M, Kawamoto F, Wataya Y et al Identification of the four species of human malaria parasites by nested PCR that targets variant sequences in the small subunit rRNA gene. Parasitol Int. 1997; 46: 91–95.

[4] Rougemont M, Van Saanen M, Sahli R, Hinrikson HP, Bille J, Jaton K. Detection of four *Plasmodium* species in blood from humans by 18S rRNA gene subunit-based and species-specific real-time PCR assays. J Clin Microbiol.2004; 42:5636–5643. doi: 10.1128/JCM.42.12.5636-5643.2004 15583293 10.1128/JCM.42.12.5636-5643.2004 PMC535226

[5] Bialasiewicz S, Whiley DM, Nissen MD, Sloots TP. Impact of competitive inhibition and sequence variation upon the sensitivity of Malaria PCR. J Clin. Microbiol. 2007; 45:1621–1623. doi: 10.1128/JCM.02145-06 17329455 10.1128/JCM.02145-06 PMC1865911

[6] Notomi T, Okayama H, Masubuchi H, Yonekawa T, Watanabe K, et al Loop-mediated isothermal amplification of DNA. Nucleic Acids Res. 2000; 28: E63 10871386 10.1093/nar/28.12.e63 PMC102748

[7] Lucchi NW, Demas A, Narayanan J, Sumari D, Kabanywanyi A, Kachur SP, et al Real-Time Fluorescence Loop Mediated Isothermal Amplification for the Diagnosis of Malaria. PLoS One. 2010; 5(10): e13733 doi: 10.1371/journal.pone.0013733 21060829 10.1371/journal.pone.0013733 PMC2966401

[8] Polley SD, Mori Y, Watson J, Perkins MD, González IJ, Notomi T, et al Mitochondrial DNA targets increase sensitivity of malaria detection using loop-mediated isothermal amplification. J Clin Microbiol. 2010; 48(8):2866–71. doi: 10.1128/JCM.00355-10 20554824 10.1128/JCM.00355-10 PMC2916605

[9] Han ET. Loop-mediated isothermal amplification test for the molecular diagnosis of malaria. Expert Rev Mol Diagn. 2013;13:205–18. doi: 10.1586/erm.12.144 23477559 10.1586/erm.12.144

[10] Oriero EC, Okebel J, Jacobs J, Geertruyden JPV, Nwakanma D, D’Alessandro U. Diagnostic performance of a novel loop-mediated isothermal amplification (LAMP) assay targeting the apicoplast genome for malaria diagnosis in a field setting in sub-Saharan Africa. Malar J. 2015; 14 (396):1–6. 26450599 10.1186/s12936-015-0926-6 PMC4599330

[11] Patel JC, Lucchi NW, Srivasta P, Lin JT, Sug-Aram R, Aruncharus S, et al Field evaluation of a real-time fluorescence loop-mediated isothermal amplification assay, RealAmp, for the diagnosis of malaria in Thailand and India. J Infect Dis. 2014; 210 (8): 1180–1187. doi: 10.1093/infdis/jiu252 24795480 10.1093/infdis/jiu252 PMC6373533

[12] Brasil. Ministério da Saúde. Sistema de Informação e Vigilância Epidemiológica da Malária. SIVEP/Malária. Available from: http://portalweb04.saude.gov.br/sivep_malaria/default.asp>. Cited 24 April 2015.

[13] Brasil. Ministério da Saúde. Secretaria de Vigilância em Saúde. Diagnóstico de Infecções Mistas—Nota Técnica nº 08/2014—CGLAB/DEVEP/SVS/MS. 2009.

[14] Singh B, Bobogare A, Cox-Singh J, Snounou G, Abdullah MS, Rahman HA. A genus- and species-specific nested polymerase chain reaction malaria detection assay for epidemiologic studies. Am J Trop Med Hyg. 1999; 60(4):687–92. 10348249 10.4269/ajtmh.1999.60.687

[15] WHO (2012). World Health Organization Global Malaria Programme. Training module on malaria control: case management Geneva 156 pp. Available from: http://www.who.int/malaria/publications/atoz/9789241503976/en/. Cited 14 May 2015.

[16] Pöschl B, Waneesorn J, Thekisoe O, Chutipongvivate S, Panagiotis K. Comparative Diagnosis of Malaria Infections by Microscopy, Nested PCR, and LAMP in Northern Thailand. American Journal of Tropical Medicine and Hygiene. 2010; (83)1, 56–60. 10.4269/ajtmh.2010.09-0630 PMC2912576 20595478

[17] Britton S, Cheng Q, McCarthy JS. Novel molecular diagnostic tools for malaria elimination: a review of options from the point of view of high-throughput and applicability in resource limited settings. Malaria Journal 15:88, 2016 doi: 10.1186/s12936-016-1158-0 26879936 10.1186/s12936-016-1158-0 PMC4754967

[18] Cavasini MTV, Ribeiro WL, Kawamoto F, Ferreira MU. How prevalent is *Plasmodium malariae* in Rondonia, Western Brazilian Amazon? Rev Soc Bras Med Trop. 2000; 33 (5): 489–492. 11064586 10.1590/s0037-86822000000500011

[19] Gamboa D, Ho M, Bendezu J, Torres K, Chiodini PL, Barnwell JW, et al A Large Proportion of *P*. *falciparum* Isolates in the Amazon Region of Peru Lack pfhrp2 and pfhrp3: Implications for Malaria Rapid Diagnostic Tests. PLoS One. 2010; 5: 1–8. 10.1371/journal.pone.0008091 PMC2810332 20111602

[20] Viana GMR, Okoth SA, Silva-Flannery L, Barbosa DRL, Oliveira AM, Goldman IF, et al Histidine-rich protein 2 (*pfhrp2*) and *pfhrp3*gene deletions in *Plasmodium falciparum* isolates from select sites in Brazil and Bolivia. PLos One. 2017; 12(3): e0171150 doi: 10.1371/journal.pone.0171150 28301474 10.1371/journal.pone.0171150 PMC5354239

[21] WHO (2005). World Health Organization. World Malaria Report. Geneva. 294 pp. Available from: http://www.who.int/malaria/publications/atoz/9241593199/en/ Cited 10 June 2006.

[22] WHO (2006). World Health Organization. World Malaria Report. Geneva. 294 pp. Available from: http://www.who.int/malaria/publications/atoz/9241593199/en/ Cited 20 April 2007.

[23] Di Santi SM, Kirchgatter K, Brunialti KCS, Oliveira AM, Ferreira SRS, Boulos M. PCR—Based diagnosis to evaluate the performance of malaria reference centers. Rev Inst Med Trop São Paulo. 2004; 46: 183–187. 15361968 10.1590/s0036-46652004000400002

[24] Lee K, Cox-Singh J, Brooke G, Matusop A, Singh B. *Plasmodium knowlesi* from archival blood films: Further evidence that human infections are widely distributed and not newly emergent in Malaysian Borneo. Int J Parasitol. 2009; 39(10): 1125–1128. doi: 10.1016/j.ijpara.2009.03.003 19358848 10.1016/j.ijpara.2009.03.003 PMC2722692

[25] Perera RS, Ding XC, Tully F, Oliver J, Bright N, Bell D, et al Development and clinical performance of high of throughput loop-mediated isothermal amplification for detection of malaria. PLos One. 2017; 12(2): e0171126 doi: 10.1371/journal.pone.0171126 28166235 10.1371/journal.pone.0171126 PMC5293265

[26] Lucchi NW, Ljolje D, Silva-Flannery L, Udhayakumar V. Use of Malachite Green-Loop Mediated Isothermal Amplification for Detection of Plasmodium spp. Parasites. PLoS One. 2016; 11(3): e0151437 doi: 10.1371/journal.pone.0151437 26967908 10.1371/journal.pone.0151437 PMC4788150

[27] Mens PF, de Bes HM, Sondo P, Laochan N, Keereecharoen L, van Amerongen A, et al Direct Blood PCR in Combination with Nucleic Acid Lateral Flow Immunoassay for Detection of *Plasmodium* Species in Settings Where Malaria Is Endemic. J Clin Microbiol. 2012, 50(11):3520–3525. doi: 10.1128/JCM.01426-12 22915610 10.1128/JCM.01426-12 PMC3486265

[28] Roth JM, de Bes L, Sawa P, Omweri G, Osoti V, Oberheitmann B, et al *Plasmodium* Detection and Differentiation by Direct-on-Blood PCR Nucleic Acid Lateral Flow Immunoassay. J Mol Diagn. 2018, 20(1):78–86. doi: 10.1016/j.jmoldx.2017.09.004 29056574 10.1016/j.jmoldx.2017.09.004

